# Supplementary material for: Impact of a Web-Based Nutrition Intervention on Eating Behaviors and Body Size Preoccupations among Adolescents
Source: Children (Basel). 2023 Oct 26;10(11):1736. doi: 10.3390/children10111736 (PMC10670448; doi:10.3390/children10111736)
Supplement: Supplementary file 1 [file children-10-01736-s001.zip › children-2641186-supplementary.pdf]

Supplementary Materials:

Supplementary Table S1. Differences in eating behavior traits between baseline and post 6-week intervention.

	Means ± SD		F-value, (*p*-value)		
			Time	Group	Interaction
**Cognitive restraint**	PRE	POST			
**Total**					
Intervention (*n* = 162)	6.6 ± 4.3	6.3 ± 4.3	2.26, (0.13)	0.52, (0.47)	0.01, (0.93)
Control (*n* = 75)	6.7 ± 3.9	6.5 ± 3.8			
**Sex**					
**Boys**					
Intervention (*n* = 56)	5.4 ± 3.9	4.9 ± 3.2	0.57, (0.45)	2.85, (0.09)	0.44, (0.51)
Control (*n* = 33)	6.2 ± 3.9	6.1 ± 3.9			
**Girls**					
Intervention (*n* = 106)	7.3 ± 4.4	7.0 ± 4.6	2.02, (0.16)	0.05, (0.82)	0.24, (0.63)
Control (*n* = 42)	7.2 ± 3.8	6.7 ± 3.7			
**Body weight status**					
**Underweight/Normal**					
Intervention (*n* = 111)	5.9 ± 3.9	5.6 ± 3.9	1.44, (0.23)	0.26, (0.61)	0.02, (0.89)
Control (*n* = 53)	5.9 ± 3.7	5.7 ± 3.8			
**Overweight/Obese**					
Intervention (*n* = 51)	8.3 ± 4.7	7.9 ± 4.8	0.83, (0.36)	0.17, (0.68)	0.00, (0.96)
Control (*n* = 22)	8.7 ± 3.7	8.2 ± 3.3			
**Disinhibition**					
**Total**					
Intervention (*n* = 162)	4.7 ± 2.5	4.8 ± 2.8	2.80, (0.10)	2.99, (0.08)	1.53, (0.22)
Control (*n* = 75)	4.7 ± 2.5	5.1 ± 2.8			
**Sex**					
**Boys**					
Intervention (*n* = 56)	4.8 ± 2.6	4.6 ± 2.6	0.17, (0.68)	0.16, (0.69)	0.31, (0.58)
Control (*n* = 33)	4.8 ± 2.6	4.8 ± 2.6			
**Girls**					
Intervention (*n* = 106)	4.7 ± 2.5	4.9 ± 2.9	5.08, (0.03)	3.80, (0.05)	1.72, (0.19)
Control (*n* = 42)	4.5 ± 2.5	5.4 ± 3.0			
**Body weight status**					
**Underweight/Normal**					
Intervention (*n* = 111)	4.7 ± 2.4	4.9 ± 2.7	2.91, (0.09)	0.82, (0.36)	0.68, (0.41)
Control (*n* = 53)	4.6 ± 2.5	5.1 ± 2.6			
**Overweight/Obese**					
Intervention (*n* = 51)	4.7 ± 2.9	4.6 ± 3.0	0.28, (0.60)	2.59, (0.11)	0.87, (0.35)
Control (*n* = 22)	4.9 ± 2.6	5.4 ± 3.4			
**Susceptibility to hunger**					
**Total**					
Intervention (*n* = 162)	5.4 ± 3.6	4.9 ± 3.6	0.68, (0.41)	0.15, (0.70)	2.44, (0.12)
Control (*n* = 75)	4.7 ± 3.6	4.8 ± 3.8			
**Sex**					
**Boys**					
Intervention (*n* = 56)	6.1 ± 3.8	4.8 ± 3.7	5.18, (0.02)	0.19, (0.67)	2.66, (0.10)
Control (*n* = 33)	5.1 ± 3.8	4.9 ± 3.8			
**Girls**					

Intervention (n = 106)	5.0 ± 3.5	4.9 ± 3.6	0.60, (0.44)	0.89, (0.35)	0.93, (0.34)
Control (n = 42)	4.3 ± 3.4	4.8 ± 3.9			
**Body weight status**					
**Underweight/Normal**					
Intervention (n = 111)	5.5 ± 3.5	5.3 ± 3.7	0.11, (0.74)	0.04, (0.85)	1.67, (0.20)
Control (n = 53)	4.8 ± 3.5	5.2 ± 4.0			
**Overweight/Obese**					
Intervention (n = 51)	5.1 ± 3.9	4.1 ± 3.3	4.66, (0.03)	0.19, (0.66)	0.66, (0.42)
Control (n = 22)	4.5 ± 3.8	4.0 ± 3.3			

Note: Mixed models for repeated measures were employed. The factors 'Group' (Intervention or Control), 'Time' (PRE or POST), and their interaction were treated as fixed effects. Participants and classes were treated as random effects to account for potential clustering. Covariates considered included Sex (Boys or Girls) and Body weight status (Underweight/Normal or Overweight/Obese).

Supplementary Table S2. Pearson's correlation between body size dissatisfaction score and changes in vegetable/fruit consumption.

	**r (*p*-Value)**
**Total**	
Intervention (n = 61)	-0.06, (0.64)
Control (n = 28)	0.62, (0.001)
**Sex**	
**Boys**	
Intervention (n = 8)	0.19, (0.72)
Control (n = 13)	0.68, (0.02)
**Girls**	
Intervention (n = 53)	-0.08, (0.58)
Control (n = 15)	0.57, (0.04)
**Body weight status**	
**Underweight/Normal**	
Intervention (n = 47)	0.02, (0.88)
Control (n = 20)	0.61, (0.008)
**Overweight/Obese**	
Intervention (n = 14)	0.16, (0.60)
Control (n = 8)	0.40, (0.43)

Note: The reduced n (sample size) in this analysis is a result of excluding adolescents with missing information on vegetable/fruit consumption in the PRE and/or POST.
Total was ajusted for age, sex and body weight;
Sex was adjusted for age and body weight;
Weight status was ajusted for age and sex.
